# Exploring metabolism flexibility in complex organisms through quantitative study of precursor sets for system outputs

**DOI:** 10.1186/1752-0509-8-8

**Published:** 2014-01-23

**Authors:** Oumarou Abdou-Arbi, Sophie Lemosquet, Jaap Van Milgen, Anne Siegel, Jérémie Bourdon

**Affiliations:** 1 IRISA UMR 6074, Université Rennes 1, Campus de Beaulieu, 35042 Rennes Cedex, France; 2 CNRS, IRISA UMR 6074, Campus de Beaulieu, 35042 Rennes Cedex, France; 3 INRIA, Campus de Beaulieu, 35042 Rennes Cedex, France; 4 INRA, UMR1348 Pegase, F-35590 Saint-Gilles, France; 5 Agrocampus Ouest, UMR1348 Pegase, F-35000 Rennes, France; 6 LINA, UMR 6241, Université de Nantes, Nantes, France

**Keywords:** Flux Balance Analysis, Flux distributions exploration, Yield variability, Nutritional model

## Abstract

**Background:**

When studying metabolism at the organ level, a major challenge is to understand the matter exchanges between the input and output components of the system. For example, in nutrition, biochemical models have been developed to study the metabolism of the mammary gland in relation to the synthesis of milk components. These models were designed to account for the quantitative constraints observed on inputs and outputs of the system. In these models, a compatible flux distribution is first selected. Alternatively, an infinite family of compatible set of flux rates may have to be studied when the constraints raised by observations are insufficient to identify a single flux distribution. The precursors of output nutrients are traced back with analyses similar to the computation of yield rates. However, the computation of the quantitative contributions of precursors may lack precision, mainly because some precursors are involved in the composition of several nutrients and because some metabolites are cycled in loops.

**Results:**

We formally modeled the quantitative allocation of input nutrients among the branches of the metabolic network (AIO). It corresponds to yield information which, if standardized across all the outputs of the system, allows a precise quantitative understanding of their precursors. By solving nonlinear optimization problems, we introduced a method to study the variability of AIO coefficients when parsing the space of flux distributions that are compatible with both model stoichiometry and experimental data. Applied to a model of the metabolism of the mammary gland, our method made it possible to distinguish the effects of different nutritional treatments, although it cannot be proved that the mammary gland optimizes a specific linear combination of flux variables, including those based on energy. Altogether, our study indicated that the mammary gland possesses considerable metabolic flexibility.

**Conclusion:**

Our method enables to study the variability of a metabolic network with respect to efficiency (i.e. yield rates). It allows a quantitative comparison of the respective contributions of precursors to the production of a set of nutrients by a metabolic network, regardless of the choice of the flux distribution within the different branches of the network.

## Background

When studying metabolism, it is important to elucidate how fluxes are distributed among the different pathways of the metabolic network with respect to the available quantitative information about the system behavior. Several methods can be used to address this issue. The first approach consists of building a mechanistic description of transformations and identifying the regulations involved in the system. Continuous dynamical models are often used for this purpose, especially when time-series responses to different treatments are available to infer the dynamics of the network. Static approaches such as Petri net can also identify qualitative distributions of fluxes in a metabolic network [1], and their stochastic extensions can even take into account stoichiometric and kinetic information [2]. With a complementary approach, one can study a system at the functional level, based on the study of fluxes at steady states. This is the purpose of the Flux Balance Analysis (FBA) framework, which has evolved considerably over the past decades [3,4]. With an FBA, the identification of external regulations is not necessary because it is assumed that the global behavior of the system can be modeled by optimizing linear combinations of selected fluxes (i.e. the *objective function*). Roughly, the methods developed in this field aim to explore a convex space of plausible flux distributions and to study the *extreme* flux distributions obtained when optimizing a linear objective function. It allows checking whether an extreme flux distribution is consistent with experimental data and to predict new experimental observations [5-8]. Nonetheless, the consistency of the solutions obtained by FBA depends on the quality of the constraints integrated in the model. To overcome this limitation, several extensions have been proposed in the literature. These extensions can be broken down into two parts. The first incorporates additional biological knowledge, such as reaction thermodynamics [9] or multioptimization [10]. The second is based on the use of FBA to globally analyze large-scale metabolic networks. For example, in flux variability analysis [11], the minimum and the maximum flux for each reaction in the network are computed under some (sub-)optimal conditions.

In this paper, our main purpose is to extend this framework to study the variability of a metabolic network at the level of *efficiencies* instead of *fluxes*. Indeed, when studying a metabolism at the organ level, a major challenge consists in comparing the efficiency, or yield rates, of two metabolic situations, i.e. the response to various input patterns. A typical example of such studies are those concerning animal nutrition, which aim to predict the quality and quantity of animal production (meat, fat, milk, etc.) in response to breeding factors. In this field, the energy and protein conversion efficiencies are derived from the study of the flux distribution of input nutrients between the different branches of the metabolic network [12]. More generally, although its definition depends on the field of application, the concept of efficiency is often linked to energy, mass growth and protein conversion [13-15]. However, the computing of efficiencies is prone to difficulties and errors, since it requires computing the quantities precursors sets which are required to explain the measured composition of outputs, although some of the internal products may be recycled within cycles [16]. With this goal, we defined the *allocation of an input towards an output* (with the abbreviation AIO) to be the proportion of a matter component (such as carbon) in a given input flux that is recovered in the selected output flux. Our definition is based on the choice of a material component, such as carbon or nitrogen, which allows a comparison of the contributions of input metabolites to the composition of output products. It can be seen as a yield rate, which is uniform among all the outputs of the system, allowing a precise understanding of the precursors used. As a first methodological contribution, we prove that AIO can be uniquely described and computed, even in the case when there are metabolic system cycles, by a matrix whose coefficients are nonlinear functions of the flux variables. Introducing nonlinearity in the definition of AIO cannot be avoided because of the presence of these cycles.

**Figure 1:**
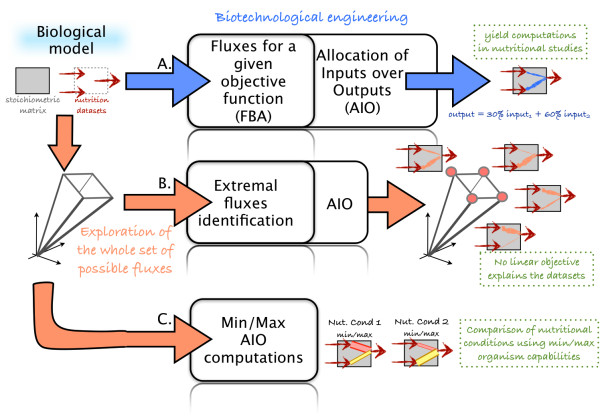
**Main functionalities of the analysis workflow.** First, extremal vertices of the simplex polyhedron of plausible flux distributions have to be computed, including the case when this space is not bounded. Then, formal algebra is required to obtain a symbolic representation of AIO matrices, expressed as a formal function, where the variables are the coefficients of a plausible flux distribution. Finally, extrema of the AIO coefficients are computed among the complete simplex of plausible flux distributions, either with the existing optimization routine or with dedicated local-search algorithms.

Studying the variability of AIO within a complete space of plausible flux distributions requires the solving of nonlinear optimization problems which are underdetermined in tangible applications. As a second methodological contribution, we have proposed efficient algorithms to compute lower and upper bounds for AIOs over the family of flux distributions which are compatible with both the system’s stoichiometry and the experimental datasets, regardless of the choice of a flux distribution for the internal branches of the network. An important aspect is that when the metabolic network is provided with input-output data, the complete space of plausible distributions appears to have a relatively small size, and can therefore be studied with our method. Our framework is depicted in Figure 1.

Our main example of application is related to milk production. In this context, several models have been introduced, in relation with the aforementioned classification of models. One class of small-size dynamical mechanistic models predicts the blood flow and input nutrients of the metabolic system (i.e. the mammary gland) [17,18] or, alternatively, the nutrients produced by the metabolic system (in terms of milk composition) [19,20]. Another class of models predicts the distribution (i.e. partitioning) of fluxes over the pathways from the input and output nutrients of the system [21,22]. In the latter family, both dynamic and static approaches exist. Indeed, numerical models based on mass-action equations were initially proposed to describe the fluxes to and from individual metabolites, with different levels of description [21,22]. To that goal, optimizers were used to determine a reasonable set of parameter estimates for the dynamical model of the system. Although such a set of parameter estimates is not unique. With a totally different approach, in another study, the main biochemical pathways in the different metabolisms were integrated in a generic stoichiometric model called the *metabolic spreadsheet*[15,23]. Its structure was based on a restricted number of intermediary metabolites called carbon-chain *pivots*, which correspond to important cross-over points between metabolic pathways. This allowed the computation of the flux rates for all reactions, constrained by a general rule stating that there is no accumulation of intermediary metabolite of cofactors. A study of manual calculations performed with the metabolic spreadsheet showed that it works, in practice, by maximizing an objective function (ATP production) in a convex solution space [24]. Therefore, this model can be considered as an application of the Flux Balance Analysis framework [25].

Nonetheless, in this field of study, there has been little discussion on the impact of the choice of a single model among several (possibly infinitely many) reasonable models [14]. To investigate this issue in a more automatic way, we compared the aforementioned conventional models to the convex space of plausible flux distributions associated with steady states of a model of mammary gland metabolism, as computed in FBA. We checked the consistency of extreme flux distributions of nutrients with experimental data of the mammary gland and milk production, including the contribution of nutrient input-output and isotope balance studies [26,27]. Based on our AIO computation framework, our analysis highlighted that the metabolic behavior of the mammary gland cannot be modeled by maximizing ATP production or by optimizing a linear combination of flux variables of the model of the mammary gland. In other words, although an infinite number of flux distributions are compatible with the data, none are extreme within the space of feasible models. Selecting any of these nonoptimal flux distributions to predict the system behavior appears difficult without additional experimentation.

To gain better understanding of the system response regardless of the choice of a flux distribution for the internal branches of the network, we applied our method to estimate the variability of AIO coefficients in our model and compared the effects of two different diets on mammary gland metabolism. Our results suggest that the bounds of AIO are sufficient to distinguish the effects of different nutritional treatments without selecting a flux distribution for the internal reactions of the metabolic network by any method - optimization of a linear combination of fluxes or a residual score. Overall, the complete study suggests considerable flexibility in mammary gland metabolism. It provides a view of the functioning of the system although its internal processes still cannot be clarified because of limitations on experimentation on large animals such as ruminants.

## Results

**Figure 2:**
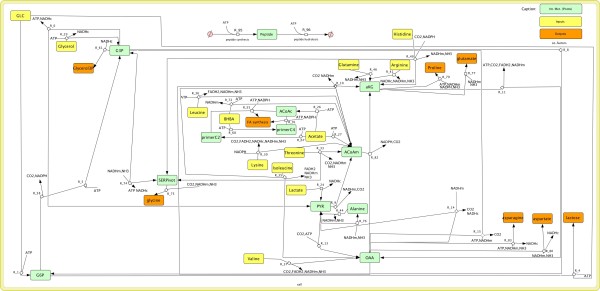
**Simplified view for the stoichiometric model of ruminant mammary metabolism with no long-chain fatty acid oxidation.** The complete model is detailed in a SBML Additional file 1. Eleven nodes are considered as pivots (green nodes), that is, intermediary metabolites which are not accumulated in the cell. The model is built by adding 26 specific reactions to the ruminant mammary to the generic model of [23]. A treatment is characterized by a set of input nodes (yellow nodes), quantitatively described in mmol/h/half udder. Nutrients contained in the milk are considered as output nodes. The node “Fatty acid synthesis” is an abstraction for 14 reactions corresponding to C(4:0), C(6:0), …C(16:0) from primerC2 and primerC4. Note that, depending on the treatment, the role of nonessential amino acids may change from input to output according to the balance of the amino-acid considered. The node “peptide” summarizes the ATP cost of protein synthesis and protein degradation. Notably, the stoichiometry of reactions was adjusted in order to balance carbon exchanges, including CO2. This is a key point in order to compute exact allocation tables in the following stages. Additional literature-based information allows us to generate additional linear constraints on some reactions fluxes.

We first investigated the set of flux distributions that are compatible with the stoichiometry of our mammary gland model (depicted in Figure 2), without taking datasets into account. The model exhibited a large variability, since thousands of extreme pathways could be identified.

We then successively computed the set of flux distributions compatible with the model and the real datasets of lactation metabolism in dairy cows. The datasets are given in Table 1. They include a control diet (Ctrl), a diet related to an increased protein supply through casein infusion into the duodenum (CN), and a complementary dataset (HB) previously used in a mechanistic model of the mammary metabolism [21]. For the three datasets, the set of plausible flux distributions – solutions to a linear system detailed in Eq. (1) – was an unbounded convex cone of dimension 5. In all cases, the solution spaces shared the same set of five independent variables. This suggests that there are five independent levels of variability within the system: peptide hydrolysis (*R*_64_), NADPH oxidation (*R*_19_), OAA →PYR (*R*_14_), OAA →G3P (*R*_15_) and G3P →G6P (*R*_8_). Therefore, by including this dataset the model became a fairly small and constrained network. Nevertheless, it was not uniquely determined since there were still several degrees of freedom.

**Table 1:** Net uptake and milk component output of the mammary gland in three treatments

	**Input or output flux**	**(Ctrl) [**[28,29]**]**	**(CN) [**[28,29]**]**	**(HB) [**[21]**]**		
		**mmol/h/ half udder**	**mmol/h/ half udder**	**mol/d/ udder**	**mmol/h/ half udder**	
*v*_2_	Glucose input ^(1)^	237	232	12.21	254	
*v*_95_	Glycerol input	5.84	5.74	0.033	0.69	
*v*_96_	Acetate input	510	462	18.42	384	
*v*_97_	BHBA input ^(2)^	84	167	7.25	151	
*v*_98_	Lactate input	0	0	0.023	0.48	
*v*_62_	3C(n:m)-acycoA+glycerol-	32.96	39.11	1.52	31.67	
	3P →*triglyceride*^(3)^ output ^(4)^					
**Fatty acid output (synthezized)**^**(5)**^						
*v*_100_	C(4:0)	10.08	11.59	0.46	9.48	
*v*_101_	C(6:0)	4.51	5.58	0.18	3.79	
*v*_102_	C(8:0)	2.23	2.87	0.10	2.06	
*v*_103_	C(10:0)	4.66	6.46	0.19	3.96	
*v*_104_	C(12:0)	4.23	6.12	0.17	3.56	
*v*_105_	C(14:0)	13.90	17.89	0.45	9.31	
*v*_106_	C(16:0)	18.82	21.44	0.64	13.40	
*v*_99_	Lactose output	73.80	83.52	3.81	79.28	
**Amino acids balance**^**(6)**^**i.e. entry or output**						
*v*_128_	Alanine input	3.11	0	0.105	2.19	Alanine catabolism
*v*_121_	Alanine output	0	3.26	0	0	Alanine synthesis
*v*_119_	Arginine input	4.40	4.48	0.526	10.96	Arginine catabolism
*v*_134_	Asparagine output	0	0	0.023	0.48	Asparagine synthesis
*v*_125_	Aspartate output	3.43	4.13	0.247	5.15	Aspartate synthesis
*v*_122_	Glutamate output	0.54	6.33	0.230	4.79	Glutamate synthesis
*v*_131_	Clutamine input	1.22	1.79	0.072	1.50	Glutamine catabolism
*v*_120_	Glycine output	4.98	3.44	0.248	5.17	Glycine synthesis
*v*_124_	Proline output	10.65	10.99	0.670	13.96	Proline synthesis
*v*_136_	Serine output ^(7)^	7.21	7.50	0.090	1.88	Serine synthesis - Serine
						used in other pathways
*v*_118_	Histidine input	0.23	0	0	0	Histidine catabolism
*v*_113_	Isoleucine input	2.19	3.57	1.518	31.63	Isoleucine catabolism
*v*_114_	Leucine input	2.02	3.76	0	0	Leucine catabolism
*v*_108_	Lysine input	2.68	3.58	0.191	3.98	Lysine catabolism
*v*_111_	Threonine input	0.35	0	0	0	Threonine catabolism
*v*_115_	Valine input	2.54	3.86	0.438	9.13	Valine catabolism
*v*_107_	Peptide output ^(8)^	124.5	150.0	7.2	149.17	
**Additional constraints**						
*v*_82_	NADPH through ICDH pathways ^(9)^	30%	30%	30%	30%	
$v_{58}=\frac{0.7}{0.3}v_{82}$	NADPH through Pentose Phosphate ^(9)^	70%	70%	70%	70%	
*v*_56_=3*v*_62_	C(n:m) →C(n:m)-acylCoA	98.87	117.32	4.56	95.00	
**FA primer from Acetate**^**(10)**^						
*v*_53_	C(4:0)	0	0	0	0	
*v*_54_=*v*_90_	C(6:0)	2.256	2.790	0.091	1.896	
*v*_55_=*v*_91_	C(8:0)	1.113	1.437	0.050	1.031	
*v*_51_=*v*_52_	C(10:0)	2.331	3.230	0.095	1.979	
*v*_86_=*v*_92_	C(12:0)	2.116	3.061	0.086	1.781	
*v*_87_=*v*_93_	C(14:0)	6.951	8.946	0.223	4.655	
*v*_88_=*v*_94_	C(16:0)	9.410	10.719	0.322	6.698	
**Other constraints**^(11)^						
*v*_24_	Lactate →Pyruvate	0	0			
*v*_44_	Alanine catabolism		0			
*v*_76_	Alanine synthesis	0		0	0	
*v*_83_	Asparagine synthesis	0	0			
*v*_40_	Histidine catabolism		0	0	0	
*v*_36_	Leucine catabolism			0	0	
*v*_33_	Threonine catabolism		0	0	0	

Data are renormalized in mmol/h/half udder. (a) Control diet *(Ctrl)*[28,29] (b) Higher protein supply by casein infusion in the duodenum *(CN)*[28,29] (c) Generic dataset *(HB)*[21]. This table is used to parameterize input and output vectors *v*_*I*_ and *v*_*O*_ together with additional biological linear constraints on some reaction fluxes.

^1^Input i.e. taken up by the stoichiometric system considered (i.e.net uptake in our example for the mammary gland).

^2^ ***β***-Hydroxybutyrate.

^3^Total triglycerides secreted in milk considering that milk fat was composed of 100% triglycerides and that all the triglycerides were secreted in milk fat [21].

^4^Output i.e. leaving the system (secreted in milk).

^5^All fatty acids synthesized within the mammary gland i.e. all C4 to C14 and 50% of C16 [21].

^6^The balance between amino acid net uptake and amino acid net output in milk protein is calculated with established rules [21,30]. If the balance is positive it corresponds to an amino acid input that will be catabolized. If this balance is negative, the values corresponded to an amino acid output (that will be synthesized to meet its requirement for milk protein).

^7^Serine output corresponded to Serine synthesized minus Serine utilized in other pathways i.e. Serine required in addition to Ser uptake to synthesize milk protein.

^8^Peptide output: number of peptide links required to synthesize the proteins exported out of the system (i.e. in milk protein).

^9^Hanigan, 1994 [21].

^10^Fatty acid primers were synthesized for 50% from acetate and for 50% from BHBA except C4 FA primer which was supposed to be synthetized only from BHBA [21].

^11^Set at zero because their inputs or outputs are set at zero (to avoid futile cycle).

### Investigating the relevance of the optimization strategies for mammary metabolism

The balance between the ATP generated by the system and the ATP used by the system (including the ATP cost of milk component synthesis) was computed for the three datasets (Ctrl), (CN) and (HB). The results are detailed in Table 2. In this table, the FBA approach based on optimization of the ATP balance is compared to the natural functioning of the system.

**Table 2:** Three different computations of ATP balance for the mammary gland in different treatments

**Biological model**	**Dataset**	**ATP balance**	**Proteic**
***Criteria of selection of a solution***			**turnover**
Mammary-gland model (Figure 3)	(HB)	6628	0
*Manual study based oncycle removal*[23]	(Ctrl)	3081	0
	(CN)	2045	0
Mammary-gland model (Figure 3)	(HB)	6628	0
*ATP optimization*	(Ctrl)	3081	0
	(CN)	2045	0
Model of Hannigan- Baldwin [21]	(HB)	4375 = 6500-2125	0
*Study of numerical equilibria of an ODE model*[31]	(Ctrl)	*Non available*	
	(CN)	*Non available*	

Three natural assumptions are considered to model the mammary gland behavior: removal of all cycles, optimization of ATP production and study of the equilibria of a dedicated ODE-based model. All models exhibit considerable variability in their ATP balance (in mmol/h/half udder), which contradicts the assumption about the behavior of this organ. Moreover, quantitatively, the computed ATP balances are much higher than recent measurements. This suggests that ATP maximization cannot be considered as a natural objective function to model cow mammary behavior.

First, we considered the manual computation of fluxes with a tool named “metabolic spreadsheet” [15,23]. We applied the rules of no accumulation on any intermediary metabolites and cofactors with a utilization rejecting all the cycles. As expected, since some cycles are ATP-consuming, both flux distributions were equivalent in both approaches (Table 2). The ATP balances were respectively 3081, 2045 and 6628 mmol/h/half udder (i.e. 318 mol/d/udder) in the (Ctrl), (CN) and (HB) datasets.

According to this model, the ATP generated is estimated to be 6500 mmol/h/half udder (i.e. 312 mol/d/udder) while 2125 mmol/h/half udder (i.e. 102 mol/d/udder) were estimated to be used for milk component synthesis [31]. Therefore, the ATP-balance obtained for this model was slightly smaller than the optimal one obtained for our mammary gland model.

Independently of the approach considered and the model or dataset at hand, the remaining ATP (ATP balance), after use for milk synthesis, appeared to be rather high and nonxconstant. Indeed, as shown in [31], an ATP balance of 1250 mmol/h/half udder (60 mol/d/udder) can be expected to be used for other functions not accounted for in the models, such as maintaining membrane potential and synthesizing nucleic acids. The numerical values obtained here were far from this estimated balance, suggesting that each of these three models have a nonrelevant ATP balance.

In addition, both in the ODE model and the ATP-optimization approach, peptide hydrolysis was obtained at zero, implying an absence of any protein turnover. This contradicts all the observations about this pathway: considerable use of this pathway has been evidenced in several publications, although the peptide hydrolysis rates differed significantly depending on the technique used for the measurements: peptide hydrolysis (*R*_64_ i.e. mammary protein degradation) spans from 0.25,0.23 to 0.67 of peptide synthesis (*R*_63_ i.e. total mammary protein synthesis) in (Ctrl), (CN) and (HB), respectively [29,31].

Overall, we concluded with this analysis that the energy-based optimization function may not allow an appropriate simulation of mammary gland metabolism. This was expected, considering that the system is studied at the complete organ level, involving competing processes which can rarely be modeled with a single linear objective function.

### Exploring all extreme flux distributions in a refined simplex

In order to study the variability within the space of plausible flux distributions and to identify alternative relevant optimization strategies, an additional constraint was placed on the ATP balance of the system. We considered that an ATP-balance of 1250 mmol/h/half udder (60 mol/d/udder) was a relevant measure for this study [31], although we checked that all the results discussed below were still valid when introducing a 10% tolerance on this estimation of ATP-balance. Providing a bound for ATP yielded a new constraint on several variables of the system. For the two datasets (Ctrl) and (CN), the flux G3P →G6P (*R*_8_) was no longer an independent variable in the system. The simplex, which was previously unbounded, appeared to be bounded with four independent variables: OAA →PYR (*R*_14_), OAA →G3P (*R*_15_), NADPH oxidation (*R*_19_), peptide hydrolysis (*R*_64_).

Applying flux variability tools [32], it appeared that the minimum of each of the fluxes *v*_14_,*v*_15_,*v*_19_,*v*_64_ was equal to zero for all treatments. The maxima of the fluxes (*v*_14_,*v*_15_,*v*_19_,*v*_64_) were (1831,1831,669,305) for the (Crtl) treatment and (795,795,22,133) for the (CN) treatment This suggests that the (Ctrl) treatment generates a more flexible space of plausible flux distributions than the (CN) treatment.

We computed all extreme vertices of the simplex of plausible flux distributions for the two treatments (Ctrl), (CN). The simplex structure of the polyhedron implies that the optimum of any linear combination of metabolic fluxes involved in the model is either uniquely attained for one of these extreme points or attained by all flux distributions positioned on a face of the simplex. To gain insight on the pathways involved in the variability of our model, we also computed the linear combination of fluxes optimized for one of the extreme flux distributions. Eight extreme vertices were found for the (Ctrl) and (CN) datasets. They were obtained when optimizing the same set of linear functions^a^. In Table 3, the optimal flux distributions for (Ctrl), (CN) are classified according to the activation or inactivation of several pathways in the model. It is worth noting that, according to this table, the two sets of plausible flux distributions associated with (CN) and (Ctrl) have the same topological structure. Indeed, the eight extremal behaviors for (CN) and (Ctrl) have clear combinatorics: first, NADPH oxidation is set either at zero or at its maximal value. Then, a single flux within OAA →PYR (*R*_14_), OAA →G3P (*R*_15_), G3P →G6P (*R*_8_), peptide hydrolysis (*R*_64_) is strongly activated whereas the three other remaining fluxes are blocked.

**Table 3:** Main properties of the simplex vertices under the assumption of constant ATP-production

	**Dataset**	**Model name**	**Example of**	**Combinatorics of pathways**					**Validation**		**Pathways with**
			**maximized**								**nonrelevant flux**
			**function**	***R***_***19***_	***R***_***15***_	***R***_***14***_	***R***_***8***_	***R***_***64***_	***R***_***63***_	***R***_***13***_	**values**
				**NADPH**	**OAA →G3P**	**OAA →PYR**	**G3P →G6P**	**Peptide**	**Peptide**	**Pyr** ***→*** **OAA**	
				**oxidation**				**hydrolysis**	**synthesis**		
Extreme flux distributions within the set of plausible solutions	(Ctrl)	B	*v*_15_- *v*_19_	0	1831	0	0	0	125	1835	*R*_13_, *R*_64_
	(CN)				795				150	803	
	(Ctrl)	F	*v*_14_- *v*_19_	0	0	1831	0	0	125	1835	*R*_13_, *R*_64_
	(CN)					795			150	803	
	(Ctrl)	D	*v*_8_- *v*_19_	0	0	0	3662	0	125	4	*R*_8_, *R*_64_
	(CN)						1590		150	8	
	(Ctrl)	H	*v*_64_- *v*_19_	0	0	0	0	305	430	4	
	(CN)							133	283	8	
	(Cntl)	A	*v*_15_ + *v*_19_	694	1714	0	0	0	125	1718	*R*_13_, *R*_64_
	(CN)			22	791				150	799	
	(Ctrl)	E	*v*_14_ + *v*_19_	694	0	1714	0	0	125	1718	*R*_13_, *R*_64_
	(CN)			22		791			150	799	
	(Ctrl)	C	*v*_8_ + *v*_19_	694	0	0	3428	0	125	4	*R*_8_, *R*_64_
	(CN)			22			1583		150	8	
	(Ctrl)	G	*v*_64_ + *v*_19_	669	0	0	0	286	410	4	
	(CN)			22				132	282	8	
Litterature-based upperbounds for fluxes							≤ 591	Non-zero	Lower than	≤ 266 mmol/h/half	
							mmol/	[28,31]	whole body	udder [33]	
							h/half udder [33]		protein synthesis [29]		

The qualitative properties of all vertices are shared in (Ctrl) and (CN) treatments. Both correspond to a simplex with height vertices. So are six of each of the (Ctrl) and (CN) simplex vertices. H and G vertices, in the (Ctrl) and (CN) treatments, are plausible with respect to the literature.

As discussed in a previous paragraph, flux distributions with no peptide hydrolysis cannot be considered as relevant [28,31]. This suggests the extreme flux distributions A-F in (CN) and (Ctrl) are not biologically relevant. On the contrary, two extreme flux distributions (distributions G and H) are consistent with the stoichiometry of the system for the (CN) and (Ctrl) treatments. These two flux distributions consist in optimizing peptide synthesis and hydrolysis after NADPH oxidation. They corresponded to a ratio between peptide hydrolysis and synthesis $\frac{v_{64}}{v_{63}}$ of 0.70 in the (Ctrl) treatment and of 0.47 in the (CN) treatment. These ratios are equal or lower to the maximum ratio of 0.67*v*_63_[31]. However, in (CN), treatment peptide synthesis was expected to be higher than in Ctrl treatments since in mammals protein synthesis is reported to increase with increasing protein intake (as CN in treatment) [29]. Curiously, on the contrary, in both flux distributions G and H, the total mammary protein synthesis decreased for the (CN) treatment when compared to the (Ctrl) treatment: *v*_63_ equals 430 or 410 mmol/h/half udder in (Ctrl) and 283 or 282 mmol/h/half udder in (CN).

### Study of quantitative contributions of precursors (AIO) for plausible extreme flux distributions

As a further investigation to check the relevance of distributions G and H in the (CN) and (Ctrl) treatments, we studied the quantitative contributions of precursors of output nutrients. This study was inspired by the usual techniques in the field of nutrition - or any domain concerned with organ studies. These techniques consist in computing yield rates to elucidate how an input nutrient may contribute to the composition of an output product, for instance to clarify what proportion of glucose, acetate or alanine taken up by the mammary gland can be recovered in the milk components (lactose, fatty acids, protein) or oxidized and recovered in CO2 released in blood. To formalize this issue, we first selected carbon as the component according to which the contributions of precursors were to be computed. Then, in order to determine how much carbon introduced into the system through a given input flux can be recovered in the rate of production of an output metabolite, we introduced a precise model for the *allocation of nutrients in measured outputs (AIO)* (see Eq. (3)). As shown in Tables 4 and 5, the nutrient allocation corresponding to both flux distributions G and H in the (CN) and (Ctrl) treatments provides evidence that glucose is the unique precursor of lactose synthesis. This contradicts studies of dairy cows suggesting that glycerol and perhaps amino acids contribute to lactose synthesis [26], and that fifteen percent of lactose carbon could not derive from glucose [27].

**Table 4:** **Origin of carbon mass within outputs for the two optimal flux distributions shown in Table **3

**Origin of carbon mass in outputs for (Ctrl) treatment**																										
**Input**	**GLC**		**Glycerol**		**Acetate**		**BHBA**		**Lys**		**Threonine**		**Isoleucine**		**Leucine**		**Valine**		**Histidine**		**Arginine**		**Alanine**		**Glutamine**	
	**Origin of the carbon mass of each output within input (in percentage of total carbon mass of each output)**																									
**Output**																										
Model	(G)	(H)	(G)	(H)	(G)	(H)	(G)	(H)	(G)	(H)	(G)	(H)	(G)	(H)	(G)	(H)	(G)	(H)	(G)	(H)	(G)	(H)	(G)	(H)	(G)	(H)
Glycerol3P	87.4	95.3	12.6	4.7	0		0		0		0		0		0		0		0		0		0		0	
lactose	100.0		0		0		0		0		0		0		0		0		0		0		0		0	
C4	0		0		0		100.0		0		0		0		0		0		0		0		0		0	
c6	0		0		66.7		33.3		0		0		0		0		0		0		0		0		0	
c8	0		0		75.0		25.0		0		0		0		0		0		0		0		0		0	
c10	0		0		80.0		20.0		0		0		0		0		0		0		0		0		0	
c12	0		0		83.3		16.7		0		0		0		0		0		0		0		0		0	
c14	0		0		85.7		14.3		0		0		0		0		0		0		0		0		0	
c16	0		0		87.5		12.5		0		0		0		0		0		0		0		0		0	
Glycine	73.0	78.3	9.2	3.7	9.7	9.8	4.0	4.1	0.3		1.4		0.3		0.2		0.3		0.8		0.4		0.2		0.1	
Glutamate	1.3	17.2	0.2	0.8	61.5	51.2	25.5	21.3	1.4	1.1	0.1		1.7	1.4	1.6	1.3	1.3	1.1	0.2	0.1	3.4	2.8	1.0	0.7	0.9	0.8
Proline	1.3	17.2	0.2	0.8	61.5	51.2	25.5	21.3	1.4	1.1	0.1		1.7	1.4	1.6	1.3	1.3	1.1	0.2	0.1	3.4	2.8	1.0	0.7	0.9	0.8
Aspartate	1.8	17.6	0.2	0.9	60.1	50.3	25.0	20.9	1.3	1.1	0.1		2.2	1.9	1.5	1.3	2.0	1.6	0.2	0.1	3.3	2.8	1.4	0.7	0.9	0.8
Peptide	0		0		0		0		0		0		0		0		0		0		0		0		0	
SERoutput	84.8	92.5	12.2	4.6	0.0		0.0		0.0		1.8		0.0		0.0		0.0		1.1		0.0		0.0		0.0	
CO2Output	37.6	35.7	0.1	1.0	38.7	39.3	16.1	16.3	1.3	1.4	0.1		1.1		1.0		1.0	1.1	0.1		1.7	1.8	0.8		0.5	

Both models have empty flux through the reactions OAA → PYR (*R*_14_), OAA → G3P (*R*_15_) and G3P → G6P (*R*_8_). Model (G) shows strong NADPH oxidation whereas model (H) has zero NADPH oxidation.

**Table 5:** **Origin of carbon mass within outputs for the two optimal flux distributions shown in Table **3

**Origin of carbon mass in outputs for (CN) treatment**																				
**Input**	**GLC**		**Glycerol**		**Acetate**		**BHBA**		**Lys**		**Isoleucine**		**Leucine**		**Valine**		**Arginine**		**Glutamine**	
	**Origin of the carbon mass of each output within input**																			
	**(in percentage of total carbon mass of each output)**																			
**Output**																				
Model	(G)	(H)	(G)	(H)	(G)	(H)	(G)	(H)	(G)	(H)	(G)	(H)	(G)	(H)	(G)	(H)	(G)	(H)	(G)	(H)
Glycerol3P	90.3	90.7	9.7	9.3	0		0	0	0		0		0		0		0		0	
Lactose	100.0		0		0		0	0	0		0		0		0		0		0	
C4	0		0		0		100.0		0		0		0		0		0		0	
c6	0		0		66.7		33.3		0		0		0		0		0		0	
c8	0		0		75.0		25.0		0		0		0		0		0		0	
c10	0		0		80.0		20.0		0		0		0		0		0		0	
c12	0		0		83.3		16.7		0		0		0		0		0		0	
c14	0		0		85.7		14.3		0		0		0		0		0		0	
c16	0		0		87.5		12.5		0		0		0		0		0		0	
Glycine	73.8	74.1	7.3	7.0	5.6		10.8		0.5		0.5		0.5		0.4		0.5		0.2	
Alanine	90.3	90.7	9.7	9.3	0		0		0		0		0		0		0		0	
Glutamate	2.5	3.0	0.2	0.3	28.7	28.6	56.1	55.8	1.6		2.4		2.5		1.7		3.0		1.2	
Proline	2.5	3.0	0.2	0.3	28.7	28.6	56.1	55.8	1.6		2.4		2.5		1.7		3.0		1.2	
Aspartate	3.8	4.3	0.4		27.8	27.6	54.2	53.9	1.6	1.5	3.2		2.4		2.6		2.9		1.2	
Peptide	0		0		0		0		0		0		0		0		0		0	
SERoutput	90.3	90.7	9.7	9.3	0		0		0		0		0		0		0		0	
CO2Output	24.2	24.1	0.2		22.2		43.4		1.9		1.8		2.0		1.7		1.9		0.8	

Both models have empty flux through the reactions OAA → PYR (*R*_14_), OAA → G3P (*R*_15_) and G3P → G6P (*R*_8_). Model (G) shows strong NADPH oxidation whereas model (H) has zero NADPH oxidation.

This precise analysis of the origin of carbon in lactose synthesis with AIO suggests that extreme flux distributions G and H have to be rejected, although both flux distributions are consistent with flux variability criteria. We concluded that none of the extreme vertices of the set of plausible flux distributions could be considered biologically relevant with respect to the model and data at hand. Notice that the optimization of any linear combination of metabolic fluxes is either reached by these extreme distributions or reached by the infinite number of flux distributions lying in a face of the simplex. This suggests that the functioning of the mammary gland cannot be uniquely modeled by the optimization of any linear combination of metabolic fluxes involved in the current model.

### Discriminate treatments despite mammary gland flexibility: maxima of AIO on the complete polyhedron of flux distributions

Previous studies suggest that the response of the mammary gland cannot be modeled uniquely by the optimization of a linear objective function of fluxes. However, there are several nonoptimal flux distributions that satisfy the literature-based information that we have used so far^b^. More generally, many flux distributions compatible with the additional constraints, including the condition on carbon precursors for lactose can be shown for both (CN) and (Ctrl) treatments. Topological arguments prove that an infinite set of flux distributions exist. Nonetheless, without an idea about the exact shape of the space of feasible fluxes (because of the nonlinear nature of the condition of carbon precursors), we cannot select a plausible point within this space. In other words, the available knowledge appears insufficient to determine uniquely a flux distribution of nutrients among the different branches of the proposed model.

To understand the functioning of the mammary gland and despite this difficulty, we introduced a method to estimate the variability of nutrient allocation among pathways on a carbon basis (see Eq. (3)) by computing the range (min-max) of AIO coefficients. As these coefficients are nonlinear functions of flux variables, computing these min and max over the complete space of plausible flux distribution requires solving nonlinear optimization problems. Our dedicated algorithm detailed in the method section scaled properly to the real case that we are studying^c^. Interestingly, with this approach, we did not favor any internal functioning of the system since we parsed all objective functions (linear combinations of flux variables), in order to have a complete description of the space of plausible flux distributions.

The min-max tables for allocations of nutrients in the different pathways provided a clearer view of nutrient utilization within the mammary gland. Unlike the functioning of the extremal distributions (G) and (H) shown in Tables 4 and 5, the contribution of carbon from glucose to lactose carbon was quite variable within the full set of plausible distribution. Indeed, as shown in Tables 6 and 7, glucose was the precursor of 85% ($\frac{1751}{886}$ or $\frac{866}{1002}$) to 100% of the carbon lactose in the (Ctrl) and (CN) treatments. Therefore, there exist flux distributions such that glucose is not the only precursor of lactose synthesis [26], and these distributions are consistent with quantitative estimations of the ratio of carbon glucose recovered in lactose [27].

**Table 6:** Minimum and maximum utilization of input in each output

**(Ctrl) treatment**																											
**Input**		**Glucose**		**Glycerol**		**Acetate**		**BHBA**		**Lysine**		**Threonine**^**1**^		**Isoleucine**^**1**^		**Leucine**^**1**^		**Valine**^**1**^		**Histidine**^**1**^		**Arginine**^**1**^		**Alanine**^**1**^		**Glutamine**^**1**^	
		**1422**		**17,5**		**1020**		**336**		**16,1**		**1,40**		**13,1**		**12,1**		**12,7**		**1,38**		**26,4**		**9,33**		**6,10**	
Output		**Minimum and maximum utilisation of Input in each output (in mmol/h/half udder of Carbon)**																									
		Min	Max	Min	Max	Min	Max	Min	Max	Min	Max	Min	Max	Min	Max	Min	Max	Min	Max	Min	Max	Min	Max	Min	Max	Min	Max
Glycerol3P	98.9	32.9	97.0	1.24	12.5	0	38.4	0	16.0	0	1.1	0	0.1	0	1.3	0	1.0	0	1.2	0	0.1	0	2.0	0	1.0	0	0.6
Lactose	886	751	886	0	7.7	0	79.8	0	33.2	0	2.3	0	0.1	0	2.6	0	2.0	0	2.4	0	0.2	0	4.0	0	2.0	0	1.1
C4	40.3	0		0		0		40.3		0		0		0		0		0		0		0		0		0	
c6	27.1	0		0		18.0		9.0		0		0		0		0		0		0		0		0		0	
c8	17.8	0		0		13.4		4.5		0		0		0		0		0		0		0		0		0	
c10	46.6	0		0		37.3		9.3		0		0		0		0		0		0		0		0		0	
c12	50.8	0		0		42.3		8.5		0		0		0		0		0		0		0		0		0	
c14	195	0		0		167		27.8		0		0		0		0		0		0		0		0		0	
c16	301	0		0		263		37.6		0		0		0		0		0		0		0		0		0	
Glycine ^2^	10.0	3.5	8.0	0.1	0.9	1.0	3.7	0.4	1.5	0.0	0.1	0.1		0.0	0.1	0.0	0.1	0.0	0.1	0.0	0.1	0.0	0.2	0.0	0.1	0.0	0.1
Glutamate ^2^	2.7	0.0	1.1	0.0	0.1	1.0	1.6	0.4	0.7	0.0	0.1	0.0	0.1	0.0	0.1	0.0	0.1	0.0	0.1	0.0	0.1	0.0	0.1	0.0	0.1	0.0	0.1
Proline ^2^	53.3	0.7	20.8	0.0	0.7	20.0	32.4	8.3	13.5	0.5	0.7	0.0	0.1	0.4	0.9	0.5	0.8	0.3	0.7	0.0	0.1	1.0	1.8	0.2	0.6	0.3	0.5
Aspartate ^2^	13.7	0.2	7.4	0.0	0.3	3.8	8.2	1.6	3.4	0.1	0.2	0.0	0.1	0.1	0.3	0.1	0.2	0.1	0.3	0.0	0.1	0.2	0.4	0.0	0.2	0.0	0.1
Serine ^2^	21.6	7.0	20.6	0.3	2.6	0.0	8.2	0.0	3.4	0.0	0.2	0.4	0.4	0.0	0.3	0.0	0.2	0.0	0.3	0.2	0.3	0.0	0.4	0.0	0.2	0.0	0.1
CO2	1126	396	565	1.3	14.5	343	446	142	185	12.1	15.3	0.7	0.8	9.1	12.4	8.7	11.3	9.1	12.1	0.7	1.0	15.1	20.3	6.3	8.8	4.2	5.6

A local-search algorithm allowed us to compute the minima and maxima of each AIO coefficient for the two treatments (Ctrl), (CN) (in mmol/h/half udder of Carbon). These tables allow discriminating the response of the mammary gland to the two treatments without requiring selection of a flux distribution for reactions in the metabolic network. (CN) treatment (protein intake by food) is characterized by a lower proportion of glucose which is oxidized in CO2 than in (Ctrl).

^(1)^Amino acid input corresponded to positive balances between amino acid net uptake and amino acid and utilization in milk protein (i.e. peptide output).

^(2)^Amino acid Output corresponded to negative balance between, amino acid net uptake and utilization in milk protein (i.e. peptide output).

**Table 7:** Minimum and maximum utilization of input in each output

**(CN) treatment**																					
**Input**		**Glucose**		**Glycerol**		**Acetate**		**BHBA**		**Lysine**^**1**^		**Isoleucine**^**1**^		**Leucine**^**1**^		**Valine**^**1**^		**Arginine**^**1**^		**Glutamine**^**1**^	
		**1392**		**17.2**		**924**		**668**		**21.5**		**21.4**		**22.6**		**19.3**		**26.9**		**8.95**	
Output		**Minimum and maximum utilisation of input in each output (in mmol/h/half udder of Carbon)**																			
		Min	Max	Min	Max	Min	Max	Min	Max	Min	Max	Min	Max	Min	Max	Min	Max	Min	Max	Min	Max
Glycerol3P	117	36.1	115	1.8	11.3	0	22.8	0	44.5	0	1.6	0	2.3	0.0	2.0	0.0	2.0	0.0	2.2	0.0	0.9
Lactose	1002	866	1002	0	9.2	0	37.9	0	74.0	0	2.6	0	3.9	0.0	3.3	0.0	3.3	0.0	3.7	0.0	1.5
C4	46.4	0		0		0		46.4		0		0		0		0		0		0	
C6	33.5	0		0		22.3		11.2		0		0		0		0		0		0	
C8	23.0	0		0		17.2		5.7		0		0		0		0		0		0	
C10	64.6	0		0		51.7		12.9		0		0		0		0		0		0	
C12	73.4	0		0		61.2		12.2		0		0		0		0		0		0	
C14	250	0		0		215		35.8		0		0		0		0		0		0	
C16	343	0		0		300		42.9		0		0		0		0		0		0	
glycine ^2^	6.9	2.1	5.5	0.0	0.5	0.4	1.3	0.7	2.6	0.0	0.1	0.0	0.1	0.0	0.1	0.0	0.1	0.0	0.1	0.0	0.1
Alanine ^2^	9.8	1.4	9.6	0.0	0.9	0	2.4	0	4.7	0	0.2	0	0.3	0	0.2	0	0.2	0	0.2	0	0.1
glutamate ^2^	31.7	0.8	8.8	0.0	0.4	6.7	9.0	13.0	17.6	0.4	0.5	0.4	0.7	0.6	0.8	0.3	0.5	0.7	0.9	0.3	0.4
proline ^2^	55.0	1.4	15.3	0.0	0.6	11.6	15.6	22.6	30.5	0.7	0.9	0.8	1.3	1.0	1.4	0.5	0.9	1.2	1.6	0.5	0.7
aspartate ^2^	16.5	0.6	7.0	0.0	0.3	2.7	4.5	5.3	8.9	0.2	0.3	0.3	0.5	0.2	0.4	0.2	0.4	0.3	0.5	0.1	0.2
serine ^2^	22.5	6.9	22.0	0.3	2.2	0	4.4	0	8.5	0	0.3	0	0.4	0	0.4	0	0.4	0	0.4	0	0.2
CO2	1021	244	402	2.0	12.5	180	227	351	444	16.3	19.8	14.2	19.1	15.8	20.0	13.4	17.4	14.8	19.2	5.9	7.8

A local-search algorithm allowed us to compute the minima and maxima of each AIO coefficient for the two treatments (Ctrl), (CN) (in mmol/h/half udder of Carbon). These tables allow discriminating the response of the mammary gland to the two treatments without requiring selection of a flux distribution for reactions in the metabolic network. (CN) treatment (protein intake by food) is characterized by a lower proportion of glucose which is oxidized in CO2 than in (Ctrl).

^(1)^Amino acid input corresponded to positive balances between amino acid net uptake and amino acid and utilization in milk protein (i.e. peptide output).

^(2)^Amino acid Output corresponded to negative balance between, amino acid net uptake and utilization in milk protein (i.e. peptide output).

More generally, the intervals of distributions of nutrients in different pathways were used to compare the effects of the (Ctrl) and (CN) treatments. Biologically, in comparison to the (Ctrl) treatment, the (CN) treatment was characterized by a lower proportion of glucose (on a carbon basis) which is oxidized in CO2, and a larger ratio used for lactose synthesis. This hypothesis can be sustained since the intervals of distribution of carbon from glucose in CO2 and lactose almost did not overlap in the (Ctrl) and (CN) treatments. More precisely, in the (Ctrl) treatment, from $\frac{396}{1422}=27.9\%$ to $\frac{565}{1422}=39.7\%$ of glucose carbon was oxidized to produce CO2, whereas this ratio ranged from 17.5*%* to 28.9*%* for the (CN) treatment. In addition, in the (Ctrl) treatment, 52.8*%* to 62.3*%* of the glucose carbon was required to produce lactose carbon, whereas 62.2*%* to 72% of glucose was required during the (CN) treatment.

Altogether, this analysis allowed us to discriminate the effects of the different treatments whatever the internal functioning of the system may be: the (CN) treatment (increase in protein supply to cows) was characterized by a lower proportion of glucose oxidized in CO2 than in (Ctrl). It appears to be a suitable strategy to analyze the metabolism flexibility without selecting a precise flux distribution or making any assumption on the internal metabolic fluxes.

As a final study, we used an interior point exploration method to estimate the AIO variability over the boundary of the simplex, rather than the complete space that was explored previously. As both tables appeared to be equal, we concluded that optimized AIO are reached on the boundary of the simplex. However, not all extreme vertices of the simplex are relevant biologically and they do not optimize AIO. This suggests that the flux distributions that optimize an AIO coefficient are placed at the interior of the simplex faces. In the future, it may be interesting to study the biological significance with multi-objective approaches [34] and check whether these can be considered as a “characteristic” point of these faces.

### Impact of long-chain fatty acids oxidation over the model predictions

As a last study, we studied the robustness of our conclusion with respect to changes in the modeling of input (net uptake) of long-chain fatty acids (LCFA), C16 and C18, in the tricarboxylic acid (TCA) cycle. In the study of the (Ctrl) and (CN) treatments, such an input of LCFA was not considered in the system since they are not synthesized by the mammary gland and we hypothesized that they are not oxidized within the mammary gland, based on isotope measurements in studies in fed lactating goats and in nonruminants [35-37]. This hypothesis was sustained by the measured carbon balance in the (Ctrl) and (CN) datasets [28], which did not require increasing the prediction of CO2 by introducing any LCFA in the TCA cycle (see also Table 8). Nonetheless, in other contexts and models, LCFA oxidation was either measured in starvation condition [38] or introduced in order balance carbon consumption and production with respect to CO2 prediction [18,20-22]. This LCFA oxidation hypothesis was for instance retained in models studying the (HB) dataset [20,21].

**Table 8:** Effect of long-chain fatty acids (LCFA) oxidization in the triacarboxylic acid (TCA) cycle over model analysis

**Main characteristics of models with different ratios of LCFA oxidized in TCA**									
**Model**		**CO2 prediction**		**ATP**	**ATP = 1250 mmol/h/half udder**				
				**optimum**	**Extreme flux distribution (see Table 9)**			**Variability of AIO coefficients**	
**Ratio of**	**Dataset**	**Predicted**	**Ratio of**		**Total number**	**Nonplausible**	**Nonplausible**	**Glucose carbon**	**Glucose carbon**
**long-chain FA**		**CO2**	**predicted CO2**			**flux values**	**AIO**	**required to**	**oxidized**
**oxidated in TCA**			**and measured CO2**					**produce lactose**	
0%	(HB)	1546	Non available	6628	Nonrelevant hypothesis [20-22]				
	(CN)	1021	99%	2045	8	6	2	[62.2 ; 72.0]	[17.5 ; 28.9]
	(Ctrl)	1126	121%	3081	8	6	2	[52.8 ; 62.3]	[27.8 ; 39.7]
10%	(HB)	1640	Non available	7395	13	13	0	[47.7 ; 62.4]	[28.8 ; 45.9]
	(CN)	1107	107%	2739	8	6	2	[59.4 ; 72.0]	[17.5 ; 32.0]
	(Ctrl)	1202	129%	3701	8	6	2	[51.6 ; 62.3]	[27.9 ; 41.2]
20%	(HB)	1756	Non available	8336	13	13	0	[46.9 ; 62.4]	[29.0; 47.0]
	(CN)	1214	118%	3607	8	6	2	[57.3 ; 72.0]	[17.5 ; 34.6]
	(Ctrl)	1298	140%	4476	Nonrelevant hypothesis: predicted CO2 is not compatible with measured CO2 [28]				
25%	(HB)	1826	Non available	8396	13	13	0	[46.5; 62.4]	[29.0; 47.5]
	(CN)	1279	124%	4128	8	6	2	[56.3 ; 72.0]	[17.6 ; 35.8]
	(Ctrl)	1355	146%	4938	Nonrelevant hypothesis: predicted CO2 is not compatible with measured CO2 [28]				
				ATP balance is too high	Extreme distributions are not biologically relevant	For plausible ratios of long-chain FA in TCA,(CN) treatment is characterized by a lower proportion of glucose (on a carbon basis) which is oxidized in CO2, and a larger ratio used for lactose synthesis.			

To study the impact of the variability of FA oxidation, a ratio of long-chain FA (10%, 20%, 25%) was introduced in the TCA cycle. For datasets (CN), (Ctrl) and (HB) which were compatible with a given ratio of LCFA oxidation, extreme flux distributions and AIO coefficients variability were studied. Our main conclusions are robust to the introduction of LCFA oxidation (Table 8). Interestingly, as shown in Table 9, the structure of the simplex generated by the (HB) diet is more complicated than the (Ctrl) and (CN) treatments, with 13 vertices for all hypotheses. This is due to the fact that *R*_15_ is highly constrained by measurements, so that this flux cannot vanish when *R*_14_ is optimized or when *R*_19_ is maximized. All the vertices for the (HB)-simplex contradict knowledge-based literature.

**Table 9:** Effect of long-chain fatty acids (LCFA) oxidization in the triacarboxylic acid (TCA) cycle over model analysis

**Main properties of the simplex vertices, assuming constant ATP-production,**												
**with different ratios of LCFA oxidized in TCA**												
	**Dataset**	**% of FA**	**Model**	**Example of**	**Combinatorics**					**Validation**		
		**oxidated**	**name**	**maximized**	**of pathways**							
		**in TCA**		**function**								
					***R***_***19***_	***R***_***15***_	***R***_***14***_	***R***_***8***_	***R***_***64***_	***R***_***63***_	***R***_***13***_	
					**NADPH**	**OAA →G3P**	**OAA →PYR**	**G3P →G6P**	**Peptide**	**Peptide**	**Pyr** ***→*** **OAA**	
					**oxidation**				**hydrolysis**	**synthesis**		
	(Ctrl)	0%				1831				125	1835	Non relevant flux values for *R*_13_, *R*_64_
		10%				2451					2455	
	(CN)	0%	B	*v*_15_- *v*_19_	0	795	0	0	0	150	803	
		10%				1489					1497	
		20%				2357					2365	
		25%				2878					2886	
	(HB)	10%				6173				150	6145	
		20%				7115					7086	
		25%				7675					7646	
	(Ctrl)	0%					1831			125	1835	Non relevant flux values for *R*_13_, *R*_64_
		10%					2451				2455	
	(CN)	0%	F	*v*_14_- *v*_19_	0	0	795	0	0	150	803	
		10%					1489				1497	
		20%					2357				2365	
		25%					2878				2886	
	(HB)	10%					6173			150	6145	
		20%					7115				7086	
		25%					7675				7646	
	(Ctrl)	0%						3662		125	4	Non relevant flux values for *R*_8_, *R*_64_
		10%						4902				
	(CN)	0%	D	*v*_8_- *v*_19_	0	0	0	1590	0	150	8	
		10%						2978				
		20%						4714				
		25%						5756				
	(HB)	10%						12289				
		20%	D1	*v*_8_- *v*_19_- *v*_14_		29	0	14172				
		25%			0			15292	0	150	0	
		10%						12289				
		20%	D2	*v*_8_- *v*_19_- *v*_15_		0	29	14172				
		25%						15292				
	(Ctrl)	0%							305	430	4	Glucose is the unique precursor of lactose synthesis (AIO)
		10%							409	533		
	(CN)	0%	H	*v*_64_- *v*_19_	0	0	0	0	133	283	8	
		10%							248	398		
		20%							393	543		
		25%							480	630		
	(HB)	10%	H1	*v*_64_- *v*_19_- *v*_14_	0	29	0	0	1024	1174	0	Non relevant flux values for *R*_63_
		20%							1181	1331		
		25%							1274	1424		
		10%	H2	*v*_64_- *v*_19_- *v*_15_		0	29		1024	1174		
		20%							1181	1331		
		25%							1274	1424		
	(Ctrl)	0%			669	1714				125	1718	Non relevant flux values for *R*_13_, *R*_64_
		10%			694	2330					2334	
	(CN)	0%	A	*v*_15_ + *v*_19_		791	0	0	0	150	799	
		10%			22	1485					1493	
		20%				2353					2361	
	(HB)	10%			1216	5961				150	5932	
		20%				6902					6873	
Extreme flux distributions within the set of plausible solutions		25%				7462					7433	
	(Ctrl)	0%			694		1714			125	1718	
		10%					2330				2334	
	(CN)	0%	E	*v*_14_ + *v*_19_	22	0	791	0	0	150	799	
		10%					1485				1493	
		20%					2353				2361	
		25%					2874				2882	Non relevant flux values for *R*_13_, *R*_64_
	(HB)	10%	E1	*v*_14_ + *v*_19_	1216	32	5929	0	0	150	5932	
		20%			1216	32	6902				6873	
		25%			1216	32	7462				6920	
		10%					6008				5979	
		20%	E2	*v*_14_ + *v*_19_- 50*v*_15_	946	0	6949				6920	
		25%					7509				7481	
	(Ctrl)	0%			694			3428		125	4	
		10%						4659				
	(CN)	0%	C	*v*_8_ + *v*_19_	22	0	0	1583	0	150	8	
		10%						2971				
		15%						4706				
		20%						5749				
	(HB)	10%	C1	*v*_8_ + *v*_19_	1216	32	0	11858			3	
		20%						13840				
		25%						14961	0	150		
		10%	C2	*v*_8_ + *v*_19_- 50*v*_15_	946	0	29	11958			0	Non relevant flux values for *R*_8_, *R*_64_
		20%						13840				
		25%						14961				
	(Ctrl)	0%			694				286	410	4	Glucose is the unique precursor of lactose synthesis (AIO)
		10%							388	513		
	(CN)	0%	G	*v*_64_ + *v*_19_	22	0	0	0	132	282	8	
		10%							248	398		
		20%							392	542		
		25%							479	629		
	(HB)	10%	G1	*v*_64_ + *v*_19_	1216	32	0	0	989	1139	3	Non relevant flux values for *R*_63_, *R*_64_
		20%							1145	1295		
		25%							1238	1388		
		10%	G2	*v*_64_ + *v*_19_- 50*v*_15_	946	0	29		996	1146	0	
		20%							1146	1296		
		25%							1247	1397		
Litterature-based upperbounds for fluxes								≤ 591	Non-zero	Lower than	≤ 266 mmol/h/half	
							mmol/	[28,31]	whole body	udder [33]		
							h/half udder [33]		protein synthesis [29]			

To study the impact of the variability of FA oxidation, a ratio of long-chain FA (10%, 20%, 25%) was introduced in the TCA cycle. For datasets (CN), (Ctrl) and (HB) which were compatible with a given ratio of LCFA oxidation, extreme flux distributions and AIO coefficients variability were studied. Our main conclusions are robust to the introduction of LCFA oxidation (Table 8). Interestingly, as shown in (Table 9), the structure of the simplex generated by the (HB) diet is more complicated than the (Ctrl) and (CN) treatments, with 13 vertices for all hypotheses. This is due to the fact that *R*_15_ is highly constrained by measurements, so that this flux cannot vanish when *R*_14_ is optimized or when *R*_19_ is maximized. All the vertices for the (HB)-simplex contradict knowledge-based literature.

**Figure 3:**
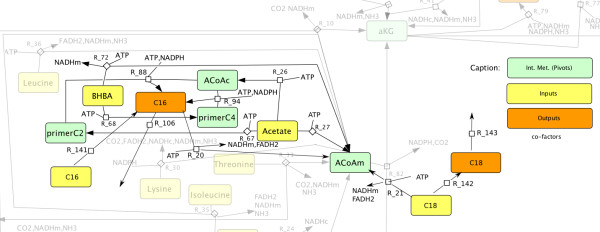
**Modeling long-chain fatty acids oxidation (LCFA) in tricarboxylic acid (TCA) cycle.** Introducing LCFA (C(16:0), C(18:0)) oxidation in the TCA cycle may be required to consistently model the response to several treatments, such as the (HB) dataset (Table 1). In this case, the model shown in Figure 2 is extended by introducing inputs of C(16:0) (*R*_141_) and C(18:0) (*R*_142_), and output of C(18:0) (*R*_143_). C(16:0) oxidation (*R*_20_) and C(18:0) oxidation (*R*_21_) are modified accordingly.

From this literature study, it appears that LCFA oxidation may depend on both the environmental and experimental contexts, and no single model can be favored yet. To study the impact of LCFA oxidation, we successively introduced a ratio of LCFA in the TCA cycle (10%, 20%, 25%), and assumed that 50% of C16 output are synthesized within the mammary gland [21] (see Figure 3). Then we performed a complete study of the (Ctrl), (CN) and (HB) datasets for these hypotheses (see Tables 8 and 9).

For the (Ctrl) treatment, by comparing the predicted and measured CO2 quantities, we concluded that the hypotheses of 20% and 25% of FA oxidized in the TCA cycle were not in agreement with our experimental data: the increase of CO2 prediction was too large both when compared to measured CO2 (see Table 8) and when confronted to the measured increase of 9% of CO2 deriving from long LCFA in lactating goats in an extreme starvation condition [38]. Similarly, the hypothesis of 0% of LCFA oxidation was not consistent with the (HB) treatment [22].

For all remaining compatible pairs of model and datasets, we first studied the ATP maximization hypothesis. In all cases, our results, shown in Table 8, suggest that ATP maximization is not biologically relevant: ATP balance was even larger than the ATP balance of the models where no LCFA was introduced in the TCA cycle (0%).

Then we enumerated extreme flux distributions and studied their biological relevance. As shown in Table 9, for each ratio of LCFA in the TCA cycle, the structure of the simplex of plausible flux distributions for the (Ctrl) and (CN) datasets was similar to that shown in Table 3 (0%). Using similar arguments, no extreme distribution could be considered as biologically relevant. When studying the space of solution associated with the (HB) dataset, a more complex structure appeared, based on 13 extreme flux distributions instead of 8. Explicit (although not unique) linear functions that can be optimized to obtain these extreme distributions are detailed in Table 9^d^. More precisely, for the (HB) treatment, we still obtain a division according to the activation of the four fluxes OAA →PYR (*R*_14_), OAA →G3P (*R*_15_), G3P →G6P (*R*_8_), peptide hydrolysis (*R*_64_). Nonetheless, an intricate phenomenon appears. Indeed, measurements imply that OAA →G3P (*R*_15_) is very constrained. It cannot vanish at the same time as OAA →PYR (*R*_14_) or when NADPH oxidation (*R*_19_) is at its maximal value. Therefore, for several optimal conditions related to NADPH oxidation (*R*_19_), OAA →PYR (*R*_14_), G3P →G6P (*R*_8_), peptide hydrolysis (*R*_64_), we need to decide whether OAA →G3P (*R*_15_) is slightly non-zero, or whether OAA →G3P is assumed to vanish although the optimal is not exactly reached for the initial flux considered. Despite this difference of structure between the (HB) dataset and the (Ctrl) and (CN) datasets, our analysis suggested that extreme distributions were not biologically relevant, independently from the ratio of LCFA oxidation or the dataset under study.

Finally, comparing the variability of the AIO coefficients for the (Ctrl) and (CN) treatments when oxidizing 10% of LCFA in the TCA cycle still suggested that the (CN) treatment is characterized by a lower proportion of glucose (on a carbon basis) which is oxidized in CO2, and a larger ratio used for lactose synthesis.

Altogether, this study suggests that the main characteristics of the (Ctrl) and the (CN) treatments are robust and could be elucidated despite lacking information on the precise internal behavior of LCFA oxidation.

## Discussions

### Analyzing the distribution of nutrients in a metabolic network to study the flexibility of a metabolism at the organ level

An important challenge in applicational fields of metabolism studies at the organ level is to understand how the components of inputs are transformed into some expected outputs, under some assumptions about the functioning of a system. To that end, great use is made of comparisons between yield rates describing the allocation of input nutrients within the set of outputs. Nonetheless, to allow a precise comparison of nutrients, these studies require insights on the distribution of matter components across the output of *each* reaction involved in the system. Such information may be provided by several experimental marking techniques (*C*13 MFA) that make use of carbon isotopes [39]. Nonetheless, their application to mammals is challenging [40].

To elucidate how input nutrients are allocated among the output nutrients of a metabolic system despite experimental limitations, we have introduced novel methods which refine the flux balance analysis of a metabolic system related to an organ of a large animal. Our method can be seen as an extension of Flux Variability Analysis [11], where the emphasize is put on *efficiency* or *yield rate* variability rather than on flux variability.

As an example of application, we have studied the mammary metabolism in ruminants (dairy cows) [15,23,24]. Compared to the conventional models available, our stoichiometric model describes very precisely the variations of energy consumptions as ATP, allowing us to investigate some optimization hypotheses related to energy variations.

As a methodological innovation, we introduced a method to estimate the *Allocation of Inputs in Outputs* (AIO), that is, the ratio of transformation of each input nutrient into outputs, provided that we are given a flux distribution balancing the production and consumption of intermediary metabolites. Two AIO computations are possible: one may fix a linear combination of flux variable to optimize, leading to an extreme flux distribution (i.e. extreme pathways) whose allocations (AIO) are precisely computed with our method. Alternatively, we can reason with regard to the possibly infinite complete convex space of plausible flux distributions, in the spirit of FVA analysis, without solving the set of equations. In this case, our method estimates the minima and maxima of each allocation (AIO) coefficient within the complete space.

The latter point is where our main algorithmic innovation lies. Indeed, AIO coefficients are not linear with respect to the flux variables *v*. Therefore, they have no reason to be extremal for flux distributions corresponding to vertices, edges or faces of the simplex, unless the gradient of every component of the AIO is a nonzero function. Therefore, we have introduced two algorithms allowing us to compute the extremal values of AIO coefficients when the flux space is bounded. The fastest algorithm requires the exhibition of an analytic expression of the AIO matrix. If this is not possible, a local-search algorithm can be used. These algorithms can be used to check whether the extremal values are reached for flux distributions lying on the boundary of the simplex of plausible flux distributions.

This approach applied to a stoichiometry model taking ATP variations into account permitted a better understanding of ruminant mammary gland metabolism in comparison to previous studies based on similar models and datasets [22,24]. For instance, we were able to characterize the differences in the effects of two treatments, such as the quantitative range of the proportion of glucose which is oxidized in CO2, or used for lactose synthesis. This information was derived from the carbon composition of each metabolite, expert knowledge which is easily accessible and is therefore compatible with the experimental limitations regarding mammals.

### Optimization strategies within tissue or organs

Our study allowed us to revisit optimization-based hypotheses on the functioning of the mammary metabolism. We have provided evidence that flux distributions corresponding to an optimal production of energy (ATP) cannot describe appropriately the metabolism of the mammary gland, as would be the case for bacterial metabolisms, which tend to optimize biomass-related functions [5,41]. The main reason is that the ATP balance is too large and variable among the responses to different treatments, confirming previous observations [31]. As a consequence, the underlying hypotheses used to drive previous studies of mammary metabolism have to be carefully reformulated [15,21,23].

As an alternative, we have hypothesized that ATP balance remains nonoptimal and almost constant in response to several treatments, and we have introduced in the model a recent estimation for this quantity [31]. Our goal was to check whether the observed responses to the system could be explained by the optimization of a linear combination of fluxes, that is, an extremal vertex of the simplex of plausible flux distributions. This hypothesis was rejected, first because it led to non-realistic orders of magnitude for some fluxes such as peptide hydrolysis, and second because the precursors of some components such as glucose were not biologically relevant [26,27]. Notably, it was necessary to trace back the quantitative origin of the nutrients to complete this analysis and reject all extreme flux distributions, illustrating the advantage of the AIO approach.

Therefore, no optimization of a linear combination of flux variables could be found to uniquely describe the metabolism of the mammary gland, a multicellular organ, as an extreme flux distribution of our model. To overcome this limitation of FBA-inspired analyses at the mammalian tissue or organ level, we studied the variability of AIO coefficients by introducing the computation of min-max ranges of AIO. This led to a general overview of the effects of treatments without precluding any steady state internal behavior.

### Benefits of studying the variability of the allocation of input in output (AIO) and future model refinements

The study of the variability of AIO, that is, intervals of allocation of nutrients, made it possible to distinguish the metabolism of the mammary gland in two different nutritional conditions corresponding to an increase in protein supply (Ctrl and CN). Notably, the intervals of allocation of glucose in CO2 and lactose were different in the two nutritional conditions, reflecting the mammary gland’s metabolic flexibility. Nonetheless, several improvements can be made to the model.

First, the simplex of plausible distributions could be reduced by introducing knowledge currently available on the kinetic bounds for enzymatic activities, introduced in previous numerical models [21]. This may for instance prevent some non-constrained behaviors shown in Tables 6 and 7, such as the possible nonzero contribution of acetate and *β*-hydroxybutyrate (BHBA) carbon to lactose. The main issue in this area will be to aggregate the enzymatic knowledge to fit with the format of the reactions in the model, such as the reaction *R*_6_ which transforms G3P to PYR under the regulation of five different enzymes, together with NAD+ and ATP availability. Therefore, proposing relevant maximal values requires the use of advanced methods for model reductions.

A second improvement of the model is dependent on the production of additional observations to clarify the set of possible behaviors of the system. Particularly, the contribution of acetate and *β*-hydroxybutyrate (BHBA) to lactose shown in Tables 6 and 7 remains questionable in ruminants since it suggests that the carbon of acetyl-COA could leave the TCA cycle of the mitochondria through the OAA → G3P pathway. This could be explained by the fact that the carbon allocation in the model did not take account of the carbon positions. However, one isotope model and one study of gluconeogenesis in man suggested that labeled carbon within glucose came from [2-14*C*]-acetate (through 14C-acetylCoA in TCA) [42,43]. It remains challenging to obtain such precise information for large ruminants such as dairy cows. Nevertheless, the model could be improved by modeling with additional constraints the few items of knowledge we have on the flux distribution of positioned carbons.

The last improvement of the model consists of including constraints that do not correspond to rule-based metabolic equations. More precisely, in several numerical models, the effects of external fluxes were introduced to predict the response of the mammary gland in a consistent way [14,17,18]. Among external fluxes, we noticed the regulatory effect of long-chain fatty acids on the activity of enzymes involved in fatty acid synthesis, although the mechanisms are not clearly understood [44]. Although some extensions of the FBA formalism to dynamic regulation exist [45], the data availability was insufficient to apply the extensions in the model. Therefore, existing regulations of enzyme activities cannot be included directly in the stoichiometric model. To overcome this limitation, we plan to investigate the effects of external fluxes by introducing additional constraints in the model, to check whether the nutrient output in the milk can be predicted from the nutrient input.

## Conclusion

We have introduced a method in the framework of flux-balance analysis of a metabolic network. As a main novelty, our approach allows studying the variability of efficiencies (or yield rates) of a metabolic model provided with input-output measurements. More precisely, our approach allows a quantitative estimation of the minimum and maximum proportions of the carbon quantity of each input nutrient which is recovered in each output component of the system. The main innovation is to propose a method which does not require determining the quantitative distribution of nutrients between the branches of the system. To that end, we have performed a parsing of the space of flux distributions which are compatible with both the model stoichiometry and input-output measurements.

This method was applied to study the response of the mammary gland to several treatments. It allowed us to distinguish two different metabolic responses of the system, corresponding to two nutritional situations and accurately reflecting metabolic flexibility. Overall, our method appears to be configured to study the variability of the yield rates of a metabolic system at the multicellular or organ level without making any hypothesis on the internal behavior of the system.

## Method

### Flux modeling based on Flux Balance Analysis

Metabolic models are described according to the generic framework of Flux Balance Analysis [6,25,46]. Metabolites split into three non-intersecting sets. First, *input metabolites* are gathered in set . The rate vector of all input fluxes (with in the form “ →*I*”) is denoted by $v_{I}\sum R^{p}$. Second, *output metabolites* are denoted by . We denote by $v_{O}\sum R^{q}$ the rate vector of the corresponding output fluxes (in the form “ *O*→”). Finally, *intermediary metabolites* (or *pivots*) are denoted by , and its cardinality is denoted by *n*. The complete set of metabolites is $ℳ=I\cup P\cup O=\left\{m_{1}\ldots m_{p+n+q}\right\}$.

Let $R=\{r_{1}\ldots r_{t}\}$ be the set of *t* reactions which produce some metabolites while degrading others. The rate vector of these reactions is denoted by $v\sum R^{t}$. A reaction *r*_*j*_ has the form $\underset{m\sum I\cup P}{\sum }s_{m,j}m\to \underset{m^{\prime }\sum P\cup O}{\sum }s_{m,j}^{\prime }m^{\prime }$ where *s*_*m*,*j*_ and $s_{m,j}^{\prime }$ are input and output stoichiometric coefficients. The metabolic system is described by the so-called *stoichiometric matrixM*=(*a*_*i*,*j*_)_*i*≤*p*+*n*+*q*,*j*≤*r*_, where $a_{i,j}=s_{m_{i},j}^{\prime }-s_{m_{i},j}$.

As a usual assumption, intermediary metabolites cannot accumulate in the cell: the consumption and production flux rates of intermediary metabolites are balanced. A linear constraint is derived on the rate vector $v\sum R^{t}$. Additional biological knowledge about the distribution of fluxes within the system is modeled by a matrix *B*. Overall, plausible flux distributions *v* satisfy the following equations:

(1) $$\begin{array}{c} M\left(\begin{array}{l} -v_{I}\\ v\\ v_{O} \end{array}\right)\;=0\;\text{[Stoichiometry constraints]};\\ \;\text{Bv}\;=0\;\text{[Biological knowledge constraints]}. \end{array}$$

Assuming that reactions are irreversible and fluxes have physical upper-bounds, the set of solutions to Eq.(1) is a convex polyhedron, called *a simplex, of plausible flux distributions*. The simplex can be described by means of its vertices, edges or faces [6,25,46], which are related to the optimization of objective functions and have shown their biological significance in several contexts [47]. In the example described below, vertices of the simplex (i.e. *extreme pathways*) were computed with standard linear-based methods in the bound case, and with probabilistic methods in the unbound case. As a refinement of these methods, we used a random sampling of the set of possible linear objective functions to obtain a complete description of the set of extreme pathways. This approach was efficient since the dimension of the space of feasible distribution is quite small, because it is strongly constrained by the input-output vectors *v*_*I*_ and *v*_*O*_.

### Modeling the quantitative contribution of input metabolites to output nutrients: AIO

In studies at the organ level such as in nutrition, the choice of a plausible flux distribution *v* aims to elucidate how an input nutrient may contribute to the composition of an output product. To formalize this issue, we introduce a *component* according to which the allocation of input nutrients will be computed. For dietary applications, carbon is a relevant element since it appears in the composition of all metabolites in the system. Nitrogen is less generic but more relevant when specifically studying nitrogen metabolism. Our issue therefore reads as follows: how much carbon introduced into the system through a given input flux can be recovered in the rate of production of an output metabolite?

We introduce the following formalism. Let $c\sum R^{p+n+q}$ be a vector describing the *component composition* of metabolites (its carbon composition for instance). Assume that the stoichiometry of each reaction in the system satisfies a matter-invariant property with respect to the component, that is: $\underset{m_{i}\sum ℳ}{\sum }a_{i,j}c(m_{i})=0$ for every reaction $r_{j}\sum R$. Of the total component mass provided as a substrate to a reaction *r*, only a part contributes to the composition of a given product *m*. Let *In*^*r*^(*m*) denote this ratio. Assuming that the system follows a *proportional matter distribution*, a consequence of the mass-invariant property is that $\underset{m\sum ℳ}{\sum }In^{r}(m)=1$ for all r∑R.

Let *v* be a plausible flux distribution and a, solution to Eq.(1). Let *m* be an input metabolite, for example glucose. The rate of the flux of component mass brought into the system by *m* equals *C*(*m*)*v*_*I*_(*m*). We denote by $x_{O}[v,m]\sum R^{q}$ the vector of proportions of input component fluxes recovered in each output flux (for instance, the proportion of carbon from the input glucose appearing in the composition of the CO2 in blood). The coefficients of *x*_*O*_[*v*,*m*] are called the *allocation of the metabolite m in output nutrients*.

To determine these ratios, we introduce $x_{P}[v,m]\sum R^{n}$ the *vector of ratios of input component fluxes* which are required to produce each intermediary metabolite *m* before its breakdown into other metabolites. The law of matter conservation and the assumption that intermediary metabolites do not accumulate put several constraints on *x*_*P*_[*v*,*m*] and *x*_*O*_[*v*,*m*], which are detailed in Tables 8 and 9. We deduce that the vector of allocations *x*_*O*_[*v*,*m*] is a solution to the equation $D_{2}[v]x_{I}^{\left(m\right)}=-D_{1}[v]\left(\begin{array}{l} x_{P}[v,m]\\ x_{O}[v,m] \end{array}\right)$ for the input parameter vector $x_{I}^{\left(m\right)}=(0,\ldots ,0,C(m)v_{I}(m),0,\ldots ,0)$, where *D*_1_ and *D*_2_ are defined in Table 10. To elucidate whether this system of matrices determines uniquely *x*_*O*_[*v*,*m*], we noticed that *D*_1_ is a square matrix such that all diagonal coefficients are equal to 1 and the others are nonpositive. This family of matrices is named the *M*-matrix in [48], and it has been shown that such a matrix is invertible. We deduce that *D*_1_ is invertible so that the vectors of allocations *x*_*O*_[*v*,*m*] and *x*_*P*_[*v*,*m*] are determined uniquely. With these formulas, when the flux distribution v has been chosen, any algorithm or method to inverse matrix *D*_1_ can be used.

**Table 10:** Modeling the quantitative allocation of input nutrients in output products

*In*^*r*^(*m*_*k*_)	=	$\left\{\begin{array}{ll} \frac{a_{k,j}C(m_{k})}{\underset{m_{i}\sum ℳ,a_{i,j}<0}{\sum }|a_{i,j}|C(m_{i})} & \text{if}\;a_{k,j}>0\\ 0 & \text{otherwise.} \end{array}\right)$	Ratio of the flux of component *c* provided as substrate to a reaction *r*_*j*_ recovered in the composition of the product *m*_*k*_. It is the sum of individual substrate contributions.
*F*(*m*_*i*_)	=	$\underset{r_{\eta }\sum R,a_{i,\eta }>0}{\sum }a_{i,\eta }v_{\eta }$ = $\underset{r_{\eta }\sum R,a_{i,\eta }<0}{\sum }|a_{i,\eta }|v_{\eta }$	Total metabolite rate involved in the production of an intermediary metabolite *m*_*i*_, before its degradation by other reactions
*d*_*k*,*i*_	=	$\left\{\begin{array}{ll} 1 & \text{if}\;k=i\\ -\underset{r_{j}\sum R,a_{i,j}<0}{\sum }\frac{|a_{i,j}|v_{j}}{F(m_{i})}*In^{r_{j}}(m_{k}) & \text{otherwise.} \end{array}\right)$	Ratio of a product flux ($m_{k}\sum P\cup O$) on the production of $m_{i}\sum I\cup P\cup O$
*D*_1_[*v*]	=	(*d*_*k*,*i*_)_*p*<*k*,*i*≤*p*+*n*+*q*_	Linear transformation of matter components contained in intermediary or output metabolites
*D*_2_[*v*]	=	(*d*_*k*,*i*_)_*p*<*k*≤*p*+*n*+*q*,1≤*i*≤*p*_	Linear transformation of matter component contained in input metabolites
*x*[*v*,*m*]	=	$\left(\begin{array}{l} x_{I}^{\left(m\right)}\\ x_{P}[x,m]\\ x_{O}[x,m] \end{array}\right)$	Rates of fluxes of component brought by the *m*-input flux appearing in the composition of each metabolites. $x_{I}^{\left(m\right)}=(0,\ldots ,0,C(m)v_{I}(m),0,\ldots ,0)$.
$D_{2}[v]x_{I}^{\left(m\right)}$	=	$-D_{1}[v]\left(\begin{array}{l} x_{P}[x,m]\\ x_{O}[x,m] \end{array}\right)$	Constraints on component fluxes deduced from thematter-invariance law, derived from Eq.(2) below.

We are given a stoichiometric matrix *A* and a vector $c\sum R^{p+n+q}$ which describes the component composition of all metabolites. If *v* is a fixed flux distribution which is compatible with the stoichiometry of the system, *AIO*[*v*] is a matrix whose (*i*,*j*) input describes the proportion of component quantity contained in *m*_*i*_ which is recovered in the flux of the output *m*.

(2) $$x[v,m](m_{k})=\underset{\begin{array}{l} \text{reactions}\\ \text{producing}m_{k} \end{array}}{\underset{︸}{\underset{r_{j}\sum R,a_{k,j}>0}{\sum }}}\underset{\begin{array}{l} \text{proportion of component}\\ \text{flux brought to the}\\ \text{composition of}m_{k}\\ \text{through}r_{j} \end{array}}{\underset{︸}{In^{r_{j}}(m_{k})}}\times \underset{\begin{array}{l} \text{total flux of component provided as substrate to}r_{j} \end{array}}{\underset{︸}{\underset{\text{substrate of}r_{j}}{\underset{︸}{\underset{m_{i}\sum ℳ,a_{i,j}<0}{\sum }}}\underset{\begin{array}{l} \text{Proportion of}\\ m_{i}-\text{component}\\ \text{consumed by}r_{j} \end{array}}{\underset{︸}{\frac{|a_{i,j}|v_{j}}{F(m_{i})}}}\times \underset{\begin{array}{l} \text{rate of the flux of}\\ \text{component}\\ \text{appearing in}\\ \text{the composition of}m_{i} \end{array}}{\underset{︸}{x[v,m](m_{i})}}}}$$

Overall, the *complete table showing the distribution of nutrient inputs in nutrient outputs* is defined to be the *q*×*p* matrix *AIO*[*v*], the columns of which are *x*_*O*_[*v*,*m*_1_], …, *x*_*O*_[*v*,*m*_*p*_], as follows:

(3) $$\begin{array}{c} \text{AIO}[v]=x_{O}[v,m].\;\;\\ \left(\begin{array}{l} x_{P}[v,m]\\ x_{O}[v,m] \end{array}\right)=-{D_{1}[v]}^{-1}D_{2}[v]\\ \;\times \left(\begin{array}{ll} C(m_{1})v_{I}(m_{1}) & \;0\\ \;\ddots \\ \;0 & C(m_{p})v_{I}(m_{p}) \end{array}\right). \end{array}$$

With these formulas at hand, as soon as the flux distribution *v* has be chosen, any algorithm or method to inverse the matrix *D*_1_ can be used. Altogether, the computation of a complete AIO table takes *O*((*n*+*p*+*q*)^3^+*np*) operations for a given flux distribution *v*.

### Computing extrema of the AIO coefficients among the complete polyhedron of plausible flux distributions: solving nonlinear optimization problems

Computing the extrema of the AIO coefficients among the convex polyhedron of plausible flux distributions *v*, leads to an optimization problem in a nonlinear context. The problem rewrites in an optimization scheme, leading to 2*q*×*p* optimization problems of the form

(4) $$\;\begin{array}{l} \forall i\leq q,\forall k\leq p,\;\text{(Max/Min)imize}\\ \left\{\text{AIO}\left[v\right]\;\left[i,,,k\right];\;v\in R^{n},\;M\left(\begin{array}{l} -v_{I}\\ v\\ v_{O} \end{array}\right)=0,\;\text{Bv}=0,\;v\geq 0\right\}. \end{array}$$

These problems are nonlinear programming (NLP) problems [49] since they aim to optimize a nonlinear objective function over a possibly nonbounded simplex search space.

When both the simplex search space was bounded and an analytical expression for *AIO*[*V*] as a function of all the free variables of the eq.(1) solution space was producible with formal algebra software [50], the Matlab *fmincon* function was used to solve the NLP problem. Among all available optimization routines, the best performance was achieved by the interior point algorithm, which has a polynomial complexity for these problems [51]. Other optimization routines were used to confirm our results, but the computation time was much longer.

When an analytical expression was not available, we implemented an alternative strategy making use of some local search routines together with the fact that *AIO*(*v*) can be computed for any *v* in a cubic time. First, we compute the Chebyshev ball *B*(*x*_0_,*r*) of the simplex [52]. Any point *x* of the *n*-dimensional ball is uniquely identified by a vector (*θ*_1_,…,*θ*_*n*_) with *θ*_*i*_∈[0,2*π*[ for 1≤*i*<*n* and *θ*_*n*_∈[0,*π*[. The intersection *P*(*x*) of the half line [*x*_0_,*x*) with the simplex of plausible flux distributions is unique if it exists. Conversely, for any point *y* on the boundary of the simplex, there exists a unique *x*∈*B*(*x*_0_,*r*) such that *y*=*P*(*x*). By extension, we say that (*θ*_1_,…,*θ*_*n*_) is a parameterization of the boundary of the simplex. This parameterization is used to determine a discretization of the simplex boundary. Given an integer *p*>0, every *θ*_*i*_ is assumed to be of the form 2*πj*/*p*, for *j*∈{0,…,*p*-1}, whereas *θ*_*n*_ rewrites as *πj*/*p*. The finite neighborhood of any point *y* on the boundary, denoted by N(y) is obtained by modifying each single coordinate of the parameterization (*θ*_1_,…,2*πj*/*p*,…,*θ*_*n*_) of *y*: for instance, among the maximal 2*n* neighbors, we find the point with paramerization (*θ*_1_,…,2*π*(*j*-1)/*p*,…,*θ*_*n*_) and (*θ*_1_,…,2*π*(*j*+1)/*p*,…,*θ*_*n*_). We can finally apply a local search algorithm based on a dichotomy principle to improve the discretization level and reach the solution to the optimization problem on the simplex boundary.

The same algorithm is extended to the full space by introducing a supplementary coordinate standing for the distance between *P*(*x*) and the center *x*_0_ of the Chebyshev ball. This makes it possible to check whether the optimum identified previously lies on the boundary of the simplex.

### Analysis workflow

A web application dedicated to the computation of AIO for metabolic networks is freely available [53]. It enables us to build a stoichiometric model, define its inputs and outputs, solve the associated FBA problem and finally compute the AIO of the selected flux distribution. Offline tools enabling the computation of the extrema of the AIO are also provided in a companion web page^e^.

The complete workflow of analysis requires the online webpage together with the use of several environments. First, *php* is required for the computation of extremal vertices of the convex polyhedron of plausible flux distributions, including the case when this space is not bounded. Second, *SAGE* is used to performed formal algebra tasks involved in the computation of AIO matrices. Finally, *matlab* is needed to compute the extrema of the AIO coefficients. The main functionalities of the workflow are depicted in Figure 2.

### Mammary gland stoichiometry model

As the basis of the ruminant mammary gland metabolism that is studied in this paper, we used a generic model of mammary metabolism [15,23]. This generic model contained 54 reactions involving six intermediary metabolites, called carbon-chain *pivots*, at cross-over points between metabolic pathways, chosen to describe the main possible conversions between metabolites: oxaloacetate (*OAA*), *α*-ketoglutarate (*α*-*KG*), pyruvate (*PYR*), acetyl coenzyme A (*AcoA*), glucose (*GLC*) and serine (*SER pivot*). The reactions also take into account the variations in ATP, four cofactors (*NADHc*, *NADHm*, *NADPH* and *FADH2*) and other metabolites (*O2*, *CO2* and *NH3*).

The model was first extended and detailed in order to include the reactions included in other models of mammary metabolism [15,21,22,24]. Reactions for lactose synthesis, milk protein, fatty acids and glycerol-3P of triglycerides were included in the model, as well as four new pivots. Mitochondrial (*mAcoA*) and cytosolic (*cAcoA*) acetyl-CoA were used to distinguish between utilization of *cACoA* for fatty acid (*FA*) synthesis and utilization of *mACoA* in the tricarboxylic acid (*TCA*) cycle. In addition, glucose-6-phosphate (*G6P*) and triose-phosphate (*G3P*) were introduced to account more specifically for the transformation of glucose through the pentose phosphate pathway, lactose synthesis and glycerol-3P utilization. For lipid metabolism, three new intermediary metabolites were introduced (*Glycerol-3P*) and two fatty acid primers (*Primer (C2:0)CoA* and *Primer (C4:0)CoA*). They correspond to the primers of two (from acetate) or four (from *β*-hydroxybutyrate; *BHBA*) carbon-units necessary to initiate fatty acid synthesis before elongation, since in ruminant mammary glands fatty acids are synthesized from acetate or *BHBA* and not glucose. Other additional reactions were included to describe milk synthesis, such as NADPH synthesis through the isocitrate dehydrogenase pathway. The input (net uptake) of long-chain fatty acids was not considered in the system since these are not synthesized by the mammary gland and we hypothesized that they are not oxidized within the mammary gland of lactating animals, as measured in well-fed goat and nonruminant through isotopes [35,37]. This hypothesis was sustained by carbon balance measurements based on experimental measures of CO2 [28]. This latter hypothesis was relaxed in a second step (see Figure 3) to study how our results are impacted by the introduction of oxidation of long-chain fatty acid within TCA cycle, as modeled in alternative contexts [18,20-22]. In addition, the action of long-chain fatty acids to regulate enzyme activities involved in C(4:0) … C(16:0) production was taken into account by the fact that C(4:0), … C(16:0) fluxes of synthesis are explicitly given in the dataset.

Protein synthesis was summarized by the number of peptide links synthesized (peptide synthesis) and the ATP required for each peptide synthesis was fixed at 5 in the present model [23]. Similarly, protein degradation was summarized by the number of hydrolyzed peptide links (peptide hydrolysis). The ATP consumption for each peptide hydrolysis was set at a single ATP [54].

Finally, four output reactions were included in the model in order to calculate the overall allocations in carbon (*R*_137_), nitrogen (*R*_138_), *CO2* (*R*_139_) and Serine (*R*_135_).

As a last step, this model was restricted to the case of ruminants. Some reactions, such as fatty acid oxidations (*R*_20_ to *R*_23_) and pyruvate synthesis through malic enzyme *R*_66_) and some input and output fluxes present in the generic stoichiometric model [23] were not considered to occur in ruminant mammary glands (fructose (*R*_25_); glucose production from *G6P* neoglucogenesis (*R*_10_), acetone synthesis (*R*_69_), *BHBA* synthesis (*R*_70_), acetoacetate synthesis (*R*_71_), and ureogenesis (*R*_50_).

Altogether, this mammary gland model contained 140 reactions, involving eleven intermediary metabolites, four cofactors and ATP, CO2, O2 and NH3. The stoichiometry of the system implies that the balance (production minus consumption) of these four last outputs can be uniquely deduced from the choice of a plausible flux distribution. The full model is shown in Figure 2 and the corresponding stoichiometric model is provided in the supplementary webpage (SBML format).

### Additional information: datasets, inputs, outputs, additional knowledge, component

Two datasets [28,29] were used, in relation to the response of dairy cow mammary gland metabolism to some increases in the protein supply: a control diet (Ctrl) and increased protein supply through casein infusion into the duodenum (CN). A complementary dataset (HB), previously used in a mechanistic model of the mammary metabolism, was used as a reference response [21]. Datasets are shown in Table 1. All data were converted into mmol/h/half udder.

Fluxes of nutrients taken up on a net basis by the mammary gland were considered as inputs whereas fluxes of nutrients produced in milk, such as lactose, milk protein, milk fatty acid arranged in triglycerides or CO2 produced and released in blood, were considered as outputs. Special attention was paid to amino acid inputs and outputs, milk protein output, fatty acids synthesized within the mammary gland and total triglyceride output according to established calculation rules [21,30]. Milk proteins were modeled by their number of peptide links (peptide output). The amino acid inputs or outputs corresponded to the balance between their real net uptake minus their contribution to peptide outputs (utilization in milk protein). When the balance of an amino acid was positive, it corresponded to an input flux. When it was negative, it corresponded to an output flux (see Table 1). Two input reactions were also assumed to be inactive in all data sets since the corresponding net uptake were not measured in the datasets (Table 1): *propionate* input (*R*_28_) and triglyceride hydrolysis (*R*_65_) in *long-chain fatty acids* input.

Eight additional linear constraints were introduced to the fluxes to ensure that the model was relevant biologically [21]. These are detailed in Tables 4 and 5 and allow building matrix *B* appearing in Eq.(1)^f^. The rate of fatty-acid acylCoA (*C(n:m)-acylCoA*) was fixed to be three times higher than the triglyceride output, assuming that 100% of milk lipids are triglycerides: it included all fatty acid acylation, that is, fatty acid synthesized in the mammary gland and long fatty acid taken up. 50% of fatty acids were assumed to be synthesized from acetate, i.e. from *primer C(2:0)CoA* and the other 50% from BHBA (*primer C(4:0)CoA*), with the exception of *C(4:0) fatty acid*, which was supposed to be synthesized only from BHBA. It was assumed that 30% of NADPH was generated by the isocitrate desydrogenase pathway and 70% of NADPH was generated in the pentose phosphate pathway [21].

*Carbon* was the component chosen to compute and analyze the allocation of nutrient inputs in nutrient outputs. The stoichiometry of the metabolic network satisfies the mass-invariant property according to this component. This required a very precise description of CO2 production and consumption in the model reactions and was validated with external measurements of the CO2 balance provided with the datasets.

## Availability of supporting data

The datasets supporting the results of this article are included within the article (Table 1). The SBML file for the mammary gland model and tools enabling the computation of all results are provided in a companion web page: http://nutritionanalyzer.genouest.org/SupplementaryMaterial.

## Endnotes

^a^ These functions are: *v*_8_+*v*_19_, *v*_8_-*v*_19_, *v*_14_+*v*_19_, *v*_14_-*v*_19_, *v*_15_+*v*_19_, *v*_15_-*v*_19_, *v*_64_+*v*_19_, *v*_64_-*v*_19_

^b^ For instance, for the (Crtl) treatment, there is a distribution such that *v*_8_<591 and *v*_13_<266 are lower than the bounds introduced in [26,27], while *v*_64_/*v*_63_=0.62<0.67 and glucose is the precursor for 90.79% of the lactose carbon

^c^ All the computations were performed by using *Matlab* release 2011 on a Xeon E5645 multicore computer.

^d^ These functions are: *v*_8_+*v*_19_, *v*_8_+*v*_19_-50 *v*_15_, *v*_8_-*v*_19_-*v*_14_, *v*_8_-*v*_19_-*v*_15_, *v*_14_+*v*_19_, *v*_14_+*v*_19_-50 *v*_15_, *v*_14_-*v*_19_, *v*_15_+*v*_19_, *v*_15_-*v*_19_, *v*_64_+*v*_19_, *v*_64_+*v*_19_-50 *v*_15_, *v*_64_-*v*_19_-*v*_14_, *v*_64_-*v*_19_-*v*_15_.

^e^http://nutritionanalyzer.genouest.org/SupplementaryMaterial. The access to the pre-formatted model and datasets supporting the results is planned to be on request. A valid access is provided by: login : SuppMat; password : tamppuss

^f^ Mathematically, these constraints can be derived as: 0.7*v*_82_-0.3*v*_58_=0; *v*_51_-*v*_52_=0; *v*_54_-*v*_90_=0; *v*_55_-*v*_91_=0; *v*_86_-*v*_92_=0; *v*_87_-*v*_93_=0; *v*_88_-*v*_94_=0; *v*_56_-3*v*_62_=0.

## Competing interests

The authors declare that they have no competing interests.

## Authors’ contributions

OA-A, JB and AS formalized the computation AIO as an optimization issue. OA-A and JB conceived the algorithms to compute AIO. OA-A performed experimentations. OA-A, SL and JV-M built the stoichiometric model. SL prepared experimental datasets and performed the biological interpretation of the results. AS and SL studied the structure on the simplex of plausible flux distributions. All authors contributed to the redaction of the paper. All authors read and approved the final manuscript.

## Supplementary Material

Additional file 1 **SBML version of the metabolic model.** The complete metabolic model of mammary gland metabolism is provided in the free and open standard SBML representation format (Systems Biology Markup Language). Click here for file
